# Supplementary material for: Assessment of a Novel Stress and Immune Gene Panel on the Development of Australasian Snapper (Chrysophrys auratus) Larvae
Source: Genes (Basel). 2024 Nov 27;15(12):1520. doi: 10.3390/genes15121520 (PMC11675413; doi:10.3390/genes15121520)

Supplementary Table S1. Showing how variables correlate with the first 5 principal components (PCs). This accounts for 79% variance in total.

	PC1 (31% variance)	PC2 (21% variance)	PC3 (12% variance)	PC4 (9% variance)	PC5 (6% variance)
**ACTB**	0.76	0.03	-0.27	0.47	0.13
**CAT**	-0.14	0.82	-0.26	0.14	-0.10
**C3-like**	0.11	-0.62	-0.01	0.12	0.26
**CRY-1**	0.10	0.76	0.39	0.31	0.02
**GSTA**	0.89	-0.23	0.09	-0.11	-0.16
**GSR**	0.47	-0.29	0.59	0.22	0.32
**GAPDH**	0.85	0.27	-0.13	-0.24	-0.26
**HSP70**	-0.46	0.53	0.18	0.55	0.22
**HSP90**	0.49	0.48	0.42	0.33	-0.30
**HAMP**	0.75	-0.22	-0.22	0.08	0.11
**UCP2-like**	0.36	0.66	0.29	-0.08	-0.20
**NRF2**	0.38	-0.01	0.62	0.15	0.38
**PRDX1**	0.82	-0.12	0.17	-0.29	-0.06
**PRDX1-like**	0.30	-0.08	-0.62	0.48	0.28
**PRDX5**	0.00	0.66	-0.17	-0.42	0.49
**TF-like**	-0.01	-0.81	-0.01	0.28	-0.07
**SOD1**	0.91	0.22	-0.05	-0.06	0.12
**PRDX3**	0.26	0.31	-0.12	-0.45	0.53

Summary

PC3 seems associated with some oscillations in the balance of GSR and NRF2 vs PRDX1-like over a 8-10 cycle; GRS and NRF2 are high or PRDX1 is low initially (Day 0 and 4), on Day 14 and on Days 22 and 24. On Days 6, 18 and 28 PRDX1 is higher and/or GRS and NRF2 are lower. PC4 seems driven by a change that occurs at Day 8, compared to Days 0-4 or Day 14 onwards; ACTB, HSP70 and PRDX1-like are low, PRDX3 and PRDx5 are higher.

Supplementary Figure S1. Images of snapper at day 2 (post hatch) and day 30.

Day 2

Gut starting to form

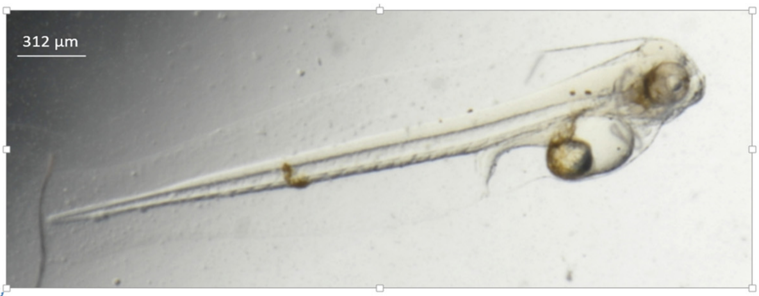

Day 30

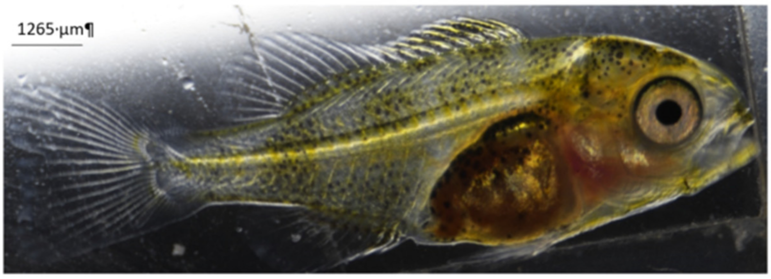

Supplement: Supplementary file 1 [file genes-15-01520-s001.zip › genes-3255834-supplementary.pdf]
